# Modeling Nonresident Seabird Foraging Distributions to Inform Ocean Zoning in Central California

**DOI:** 10.1371/journal.pone.0169517

**Published:** 2017-01-25

**Authors:** Anna J. Studwell, Ellen Hines, Meredith L. Elliott, Julie Howar, Barbara Holzman, Nadav Nur, Jaime Jahncke

**Affiliations:** 1 Romberg Tiburon Center for Environmental Studies, San Francisco State University, Tiburon, CA, United States of America; 2 Department of Geography and Environment, San Francisco State University, San Francisco, CA, United States of America; 3 Point Blue Conservation Science, Petaluma, CA, United States of America; Sveriges lantbruksuniversitet, SWEDEN

## Abstract

Seabird aggregations at sea have been shown to be associated with concentrations of prey. Previous research identified Central California as a highly used foraging area for seabirds, with locally breeding seabirds foraging close to their colonies on Southeast Farallon Island. Herein, we focus on nonresident (i.e. non-locally breeding) seabird species off of Central California. We hypothesized that high-use foraging areas for nonresident seabirds would be influenced by oceanographic and bathymetric factors and that spatial and temporal distributions would be similar within planktivorous and generalist foraging guilds but would differ between them. With data collected by the Applied California Current Ecosystem Studies (ACCESS) partnership during cruises between April and October from 2004–2013, we developed generalized linear models to identify high-use foraging areas for each of six nonresident seabird species. The four generalist species are *Phoebastria nigripes* (black-footed albatross), *Ardenna griseus* (sooty shearwater), *Ardenna creatopus* (pink-footed shearwater), and *Fulmarus glacialis* (northern fulmar). The two planktivorous species are *Phalaropus lobatus* (red-necked phalarope) and *Phalaropus fulicarius* (red phalarope). Sea surface temperature was significant for generalist species and sea surface salinity was important for planktivorous species. The distance to the 200-m isobath was significant in five of six models, Pacific Decadal Oscillation with a 3-month lag in four models, and sea surface fluorescence, the distance to Cordell Bank, and depth in three models. We did not find statistically significant differences between distributions of individual seabird species within a foraging guild or between guilds, with the exception of the sooty shearwater. Model results for a multi-use seabird foraging area highlighted the continental shelf break, particularly within the vicinity of Cordell Bank, as the highest use areas as did Marxan prioritization. Our research methods can be implemented elsewhere to identify critical habitat that needs protection as human development pressures continue to expand to the ocean.

## Introduction

Seabird distributions have been used as a basis to designate, propose, and identify marine protected areas (MPAs) around the world [1]. To designate MPA boundaries for Namibia’s first marine protected area, researchers considered the foraging range of the endangered African penguin (*Spheniscus demersus*) and later used data to confirm that the penguin’s foraging range fell within the established boundaries [2]. A fishery exclusion zone was also created and monitored based on the foraging ranges of African penguins [3]. In the European Union, important use areas for the Manx shearwater (*Puffinus puffinus*), Atlantic puffin (*Fratercula arctica*), and the lesser black-backed gull (*Larus fuscus*) were used to propose Special Protection Area boundaries that add to an existing network of conservation and protection areas [4,5]. Species-specific models of habitat of marine apex predators, coupled with climate change projections, have been used to identify some of the most threatened ecosystems at the scale of the North Pacific [6]. Seabird habitat research at the scale of the eastern North Pacific’s California Current region has highlighted the importance of the Gulf of the Farallones region off Central California [7].

Species distribution modeling can be used to identify productive marine areas for conservation [1,8–10]. Using species distribution models based on environmental conditions to identify seabird foraging areas provides a more complete picture of seabird spatial and temporal use of areas beyond sampled locations. For example, species distribution modeling results for a study conducted in the Gulf of the Farallones region off Central California showed that the distributions of multiple central-place-foraging seabirds [11] (hereafter called ‘breeding’ species) were significantly associated with their island colonies [12]. In contrast, species distribution models confirmed that distribution of a seabird visiting from its Hawaiian breeding grounds, the black-footed albatross (*Phoebastria nigripes*), was significantly associated with the 200-m isobath in this region [13]. However, no studies have taken a multispecies approach to assess critical foraging area for nonresident, non-locally breeding species (hereafter referred to as ‘nonresident species’) in this region. Foraging areas are likely to differ between breeding and nonresident species because nonresident seabirds are not constrained to local islands as are breeding seabirds [14].

The Gulf of the Farallones region encompasses productive waters of the California Current, a cold-water system spanning from southern British Columbia to Baja California [7,15]. Between the months of April and June, strong seasonal upwelling drives primary productivity that, in turn, supports a prey base that attracts numerous seabird species to the region [7]. This wind-driven upwelling is affected by regional and basin-scale climate at multiple time scales [16]. Interdecadal fluctuations of basin-wide climate indices have been linked to population impacts for seabirds breeding on Pacific islands [17]. The Pacific Decadal Oscillation (PDO) and the North Pacific Gyre Oscillation (NPGO) influence ocean conditions primarily at the multidecadal scale [18,19]. The Southern Oscillation Index (SOI) reflects ocean conditions at the interannual scale [20]. A regional Upwelling Index (UI) influences ocean conditions at daily to seasonal scales. Variability as measured by these climate indices manifests in changes to the local oceanography and influences the productivity [18,20,21] and consequently the prey base to which seabird species respond [22].

Prey species respond differently to environmental features and fluctuations, consequently influencing their predators’ foraging behavior. Marine prey such as zooplankton and forage fish exhibit patchiness over multiple spatial scales [23,24]. Where seabirds forage is indicative of where prey species occur, although not always in highest abundance [23,25]. Distinguishing spatial differences among distributions of seabird foraging guilds has been used to suggest where different prey types are available [23,26]. Here we describe species patterns of 1) generalist and 2) planktivorous foraging guilds off Central California. We use ‘generalist’ to refer to species that eat primarily a diet of fish and squid but may also include krill and offal, while we use ‘planktivorous’ to refer to species that eat almost exclusively planktonic organisms such as krill and copepods [27]. We chose common generalist species not known to breed off Central California: the sooty shearwater *(Ardenna griseus)*, pink-footed shearwater *(Ardenna creatopus*), northern fulmar *(Fulmarus glacialis*), and black-footed albatross *(Phoebastria nigripes*), as well as common planktivorous species including the red phalarope *(Phalaropus fulicarius*) and the red-necked phalarope *(Phalaropus lobatus*) [28].

Here we test the hypothesis that nonresident seabird distributions in the Greater Farallones National Marine Sanctuary (GFNMS) and Cordell Bank National Marine Sanctuary (CBNMS) are influenced by oceanographic and bathymetric features that enhance foraging opportunities at sea and not by their distance to islands or to the mainland with which breeding seabirds are generally associated. Our second hypothesis was that generalist and planktivorous species would forage in different areas in GFNMS and CBNMS. To address our hypotheses, we used a 10-year dataset to 1) identify environmental predictors that influence seabird distributions, 2) generate species distribution models using significant predictor variables to predict seasonal and annual high-use foraging area, and 3) statistically compare the predicted spatial distributions of seabird species and foraging guilds. Finally, we used our predicted species distributions to illustrate three conservation scenarios by targeting multi-species foraging areas for protection using a conservation prioritization software.

## Methods

### Study Area

**Fig 1:**
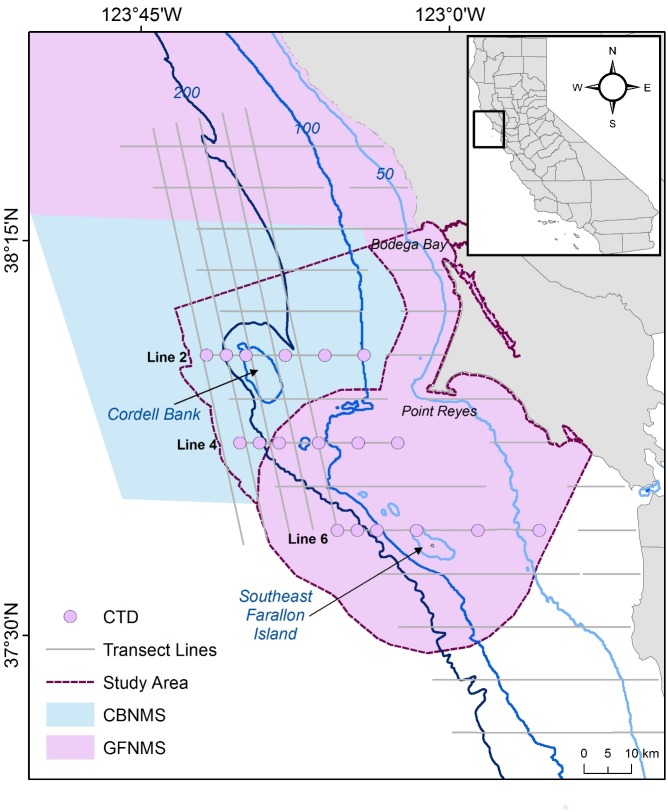
Study area. The study area encompassing ACCESS transect lines and Conductivity, Temperature, and Depth (CTD) deployment stations on lines 2, 4, and 6 within the sanctuary boundaries of CBNMS and GFNMS.

The study area (Fig 1) included the CBNMS and GFNMS off the coast of Central California. The study area encompassed the southern portion of each sanctuary (CBNMS: 1370 km^2^, GFNMS: 3320 km^2^) ranging from north of Cordell Bank (38°8’N) to south of the Farallon Islands (37° 34’N) and extending from the upper continental slope to coastal waters within 15 km from shore.

### Data Collection and Processing

This study used ten years of field survey data collected by the Applied California Current Ecosystem Services (ACCESS) team, a partnership between GFNMS, CBNMS, and Point Blue Conservation Science (www.accessoceans.org). Between April and October, 2004–2013, 37 cruises were completed. Observers followed the ACCESS standardized strip-survey design and sampling methodology (see Jahncke et al. 2008 [29] for further details). Transect lines spanned nearshore and offshore areas along the continental margin (Fig 1). We used seabird counts, detection variables, and oceanographic information collected continuously from ACCESS transects as well as bathymetric, distance, and climate data from the sources detailed in Table 1.

**Table 1:** Environmental variables. Variables used to analyze seabird distribution in GFNMS and CBNMS per 3-km survey bin for 37 ACCESS cruises from 2004–2013.

***Variable***	*Mean ± SD*	*Min−Max values*	*Description*	*Data Source*
***Oceanography***				*** ***
SSF	2.5 ± 6.7	−8.6−28.1	Avg. sea surface fluorescence (rfu) (*in situ*)	ACCESS Survey Strip Transect (1–6 meters from surface)
SSS	33.4 ± 0.36	29.7−34.1	Avg. sea surface salinity (mg/m³) (*in situ*)	ACCESS Survey Strip Transect (1–6 meters from surface)
SST	12.3 ± 1.7	8.2−16.4	Avg. sea surface temperature (°C) (*in situ*)	ACCESS Survey Strip Transect (1–6 meters from surface)
***Bathymetric Features***				*** ***
Distance to Cordell Bank	37.6 ± 24.9	0.8−115.5	Distance from bin midpoint to Cordell Bank midpoint (km)	See Source for Average Depth
Distance to 200-m isobath	11.6 ± 10.3	0.02−47.3	Distance from bin midpoint to 200-m isobath (km)	See Source for Average Depth
Depth	179.2 ± 240.0	7.6–2075.6	Avg. depth (m)	Department of Fish and Wildlife. Available from: ftp://ftp.dfg.ca.gov/R7_MR/BATHYMETRY/ (Accessed March, 2014).
Contour Index	0.2 ± 0.2	0−1	(max depth—min depth)/max depth per bin	See Source for Average Depth
***Terrestrial Features***				*** ***
Distance to mainland	26.5 ± 10.5	1.2−50.5	Distance from bin midpoint to nearest mainland (km)	United States Census Bureau. Available from: http://www.census.gov/geo/maps-data/data/cbf/cbf_counties.html (Accessed March, 2014).
Distance to island	25.9 ± 16.4	0.1−89.4	Distance from bin midpoint to nearest island (km)	United States Census Bureau. Available from: http://www.census.gov/geo/maps-data/data/cbf/cbf_counties.html (Accessed March, 2014).
***Climate Indices***				*** ***
UI	183.7 ± 71.3	46−339.5	Monthly Upwelling Index Value	Pacific Fisheries Environmental Laboratory. Available from: http://www.pfeg.noaa.gov/products/PFEL/modeled/indices/upwelling/NA/data_download.html (Accessed March, 2014).
NPGO	0.6 ± 0.9	−1.7−2.1	Monthly North Pacific Gyre Oscillation Value	Emanuele DiLorenzo. Available from: http://eros.eas.gatech.edu/npgo/ (Accessed March, 2014).
PDO	−0.4 ± 1.0	−2.2−1.9	Monthly Pacific Decadal Oscillation Value	Joint Institute for the Study of the Atmosphere and Ocean. Available from: http://jisao.washington.edu/pdo/PDO.latest (Accessed March, 2014).
SOI	0.4 ± 1.6	−2.7−4.3	Monthly Southern Oscillation Index Value	CGD University Corporation for Atmospheric Research. Available from: http://www.cgd.ucar.edu/cas/catalog/climind/soi.html (Accessed March, 2014).
***Detection Variables***				*** ***
Sea State	2.8 ± 1.4	0−6	Observed Beaufort Scale conditions (*in situ*)	ACCESS Survey Strip Transect
Swell	1.8 ± 4.0	0−8	Observed swell height (m) (*in situ*)	ACCESS Survey Strip Transect
Visibility	5.3 ± 2.0	0−6	Observed visibility (*in situ*)	ACCESS Survey Strip Transect
Cloud Cover	4.7 ± 3.4	0–8, 9 = sky obscured	Observed cloud cover (*in situ*)	ACCESS Survey Strip Transect
Time of Day	12:19:00 ± 03:00	06:08–20:05	Time of completion (hh:mm)	ACCESS Survey Strip Transect

Prior to analysis, data were processed so that values were assigned to the midpoint of each 3-km transect line segments, referred to as ‘bins’ [7,30] and to each centroid of a 1-km^2^ prediction grid cell [31,32]. For each 3-km bin midpoint and each 1-km^2^ prediction grid cell centroid (extending up to 5 km beyond the boundaries of the Sanctuaries), we extracted data on a cruise-by-cruise basis. Binned data were used to generate detailed predictive models while the gridded data provided a matrix for which models were used to predict values for the unsampled space of the study area. We used *in situ* oceanographic data for temperature, salinity, fluorescence, and detection variables (sea surface swell, visibility, Beaufort scale); we extracted bathymetric data for depth; and we calculated distances to the 200-m isobath, California mainland, the closest point of the Farallon Islands, and the midpoint of Cordell Bank. We assigned to each bin and grid centroid monthly values for all climate indices and the UI.

Seabird observations and detection variable values were recorded from the flying bridge of a research vessel using standardized strip transect methods following transect lines in the study area [29]. Surveys were conducted on one of three research vessels of varying size listed from smallest to largest: the *John H*. *Martin* (Moss Landing Marine Laboratories, Moss Landing, California, USA), the *Fulmar* (Monterey Bay National Marine Sanctuary, Monterey, California, USA), and the *McArthur II* (National Oceanic and Atmospheric Administration, Seattle, Washington, USA). Strip width was dependent on the survey vessel: Martin (~100-m), Fulmar (~200-m) and McArthur II (~300-m). Strip widths of <100 m reflect adjustments for poor visibility conditions. While traveling at a speed of 10 knots, an expert observer stationed on the flying bridge recorded seabird presence and behavior within a 90° arc from the bow to one of the vessel’s sides within 50–300 m. If conditions led to glare, the observer switched sides of the vessel. We only used records of seabirds foraging, feeding, or sitting on the sea surface to represent foraging behavior [12,33]. We assumed all seabirds exhibiting these behaviors were either actively searching for food at or near the sea surface (foraging), had prey visible in the mouth (feeding), or had just finished feeding and were resting to digest (sitting on the sea surface). We excluded records of flying seabirds because we assumed they were flying to a foraging area elsewhere and not foraging in that particular location of our study area. While underway, observers also recorded changes in the detection variables of sea state (Beaufort scale), swell height, cloud cover, and visibility. These methods have been used previously to survey seabirds off California [12].

A thermosalinograph (TSG) positioned in the sea chest of each ship recorded continuous underway measurements for sea surface temperature (SST), salinity (SSS), and fluorescence (SSF) at the sea surface. A Sea-Bird Electronics SBE 19Plus SEACAT Conductivity-Temperature-Depth (CTD) Profiler equipped with a WETStar fluorometer was deployed at designated sampling stations (Fig 1) to collect the same three oceanographic variables at the sea surface (1–6 m depth). Instruments were calibrated annually by the manufacturer. To extend our observations to within and up to 5 km beyond the larger study area, we interpolated surface water maps on a cruise-by-cruise basis from binned oceanographic data using ordinary kriging (ESRI v. 10.2) [34]. We were interested in capturing oceanographic trends (n = 107 total surfaces for SSF, SSS, and SST) in a patchy environment for which kriging has been shown to produce accurate estimates [35–37]. Using kriging, we generated prediction surfaces for each oceanographic variable and for each cruise. We evaluated surfaces based on plots of the average standard error and detrended surfaces as necessary to minimize errors across all surfaces. We used surfaces with an average standard error that fell within three standard deviations of the mean of all surfaces. One cruise (n = 3 surfaces) was excluded due to limited spatial coverage, and we only included extracted values to the region south of ACCESS transect line 3 for two others (n = 6 surfaces).

Additional processing was required for oceanographic data as TSG malfunction led to missing sea surface values for some cruises. For seabird bins that didn’t have corresponding TSG data, the closest CTD stations (within a 3-km radius of missing record locations) were used to derive raster surfaces (*n =* 23 of the 107 total oceanographic surfaces) from which we extracted values to make local predictions. We used a simple linear model regressing TSG values on extracted CTD values while controlling for month and year. Our linear models predicted (*p* < 0.001) average fluorescence (R^2^ = 0.63), salinity (R^2^ = 0.88), and temperature (R^2^ = 0.94) to fill in missing values.

In addition to our *in situ-*collected data, we also included bathymetric and distance-related factors. Previous studies have pointed to accessibility to the mainland, islands, Cordell Bank, and the 200-m isobath as being potentially important to nonresident seabird distributions [7,12]. The shortest distance from the midpoint of each 3-km bin and from the centroid of each grid cell to the nearest part of each geographic feature and depth were calculated using a geographic information system (ArcGIS v. 10.2, Environmental Systems Research Institute, 2014). Average depth was calculated from a bathymetric surface and contour index (a metric of ocean floor complexity) was derived using the following equation described in previous research: (maximum depth–minimum depth)/maximum depth [30].

To account for climatic influences on seabird distribution, we included four climate indices known to influence the California Current System: NPGO, PDO, SOI, and UI. Values for each variable were matched with cruises by month. NPGO [18], PDO [21], and SOI [38] have been characterized as basin-wide indices while UI was calculated regionally (Table 1). To represent upwelling for this study region, we used an average monthly value for two locations along the California coastline (Big Sur, 36°N 122°W and Point Arena, 39°N 125°W) as no single value matched the Sanctuaries’ region. To see if a delayed effect could influence seabird population distribution, lagged values of 1, 2, and 3 months were also calculated for climate and upwelling indices [39].

### Species Distribution Modeling

We used negative binomial regression and zero-inflated negative binomial regression to model the distribution of six nonresident foraging species [40]. All seabird species had a large proportion of zero count bins relative to non-zero count bins (Table 2). We only selected species with at least 100 non-zero bins for the entire dataset to allow for adequate model fitting, as fewer than this amount of counts often resulted in models that failed to converge [12].

**Table 2:** Description and counts of seabirds in the dataset.

*Description*	*Mean ± SD*	*Min−Max values*	*Non-zero Bins (n = 4073 total)*
*Phoebastria nigripes* (black-footed albatross)*	2.1 ± 2.3	1−20	229
*Fulmarus glacialis* (northern fulmar)*	3.9 ± 10.7	1−109	210
*Ardenna creatopus* (pink-footed shearwater)*	4.0 ± 7.1	1−50	192
*Phalaropus fulicarius* (red phalarope)†	6.7 ± 11.8	1−70	100
*Phalaropus lobatus* (red-necked phalarope)†	7.8 ± 12.1	1−86	174
*Ardenna griseus* (sooty shearwater)*	35.9 ±104.3	1−1405	651

Foraging type depicted by

* (generalist) and

† (planktivorous).

Non-zero bins are the number of bins in the dataset that contained a sighting of one or more individuals of that species.

### Univariate Model Selection

Using negative binomial regression (STATA 13, StataCorp. College Station, TX), we developed a systematic approach to selecting the most significant relationship of each variable to include as a covariate in a later multivariable model. For all models, we controlled for month and year as respective quadratic and categorical variables and used the natural log of the binned areas as an offset to account for seabird detection differences that result from bins with different areas [40,41]. We used the likelihood ratio test of alpha (the over-dispersion parameter) to determine whether negative binomial models were preferable to Poisson regression.

For oceanographic (SSF, SSS, SST) and bathymetric variables (average depth and contour index), we compared linear or quadratic relationships tested in a univariate regression. Significant quadratic variables (*p* < 0.05 for the quadratic term) were always included, but if the quadratic term was not significant, the linear variable was included (provided *p* < 0.20). For distance variables (distance to: 200-m isobath, mainland, nearest island, and Cordell Bank), we selected the most significant relationship of linear, quadratic, logarithmic, or inverse logarithmic relationships. Climate conditions prior to the time of observation ranging from daily to interdecadal time scales may influence seabird foraging distributions [7]. Seabird data were aggregated to a monthly temporal scale enabling comparison of the present conditions (no lag) as well as 1-, 2-, and 3-month lag periods for each climate index (NPGO, PDO, SOI, and UI). We assumed that including up to a 3-month lag would capture the influence of climate drivers on the oceanography of this region [42,43]. We chose from either linear or quadratic relationships, only including the lag and functional relationship that were most significant.

### Multivariable Model Selection

All significant variables from the univariate models were included in a preliminary multivariable model. We controlled for month and year and included the natural log of the binned survey area as an offset. We used manual backwards stepwise removal until all variables were significant (*p* < 0.05). We used the variance inflation factor (VIF) statistic to test for multicollinearity and ensured that all VIF values were below 10 [44,45].

To allow for the effect of covariates to vary among years [7], we examined the possible change in the effects of local and regional predictors over time. We tested for interactions of significant local and regional covariates, specifically the interaction of depth, the 200-m isobath, SST, and UI each with year (as a categorical variable). If more than one interaction was significant (*p* < 0.05), we included all interactions in the model.

We accounted for potential “false zeroes” (i.e. failure to detect individuals that are present) [14,41,46] by evaluating whether zero-inflated negative binomial models outperformed the conventional negative binomial models. We used the Vuong test statistic to determine if the zero-inflated negative binomial version was preferable to the standard negative binomial model [47]. If more than one detection variable was significant, all significant detection variables were included in the final model.

### Model Validation and Interpretation

We validated model fit using k-fold cross validation, repeating cross validation (k = 10) 20 times after randomization to validate each model’s fit [42]. For k-fold validation, each 1/k subset, in this case 10%, of the binned data was held out and its values were predicted from the remaining 90% of the data [42,48]. A median pseudo R^2^ value was calculated for each of the 20 runs and a median of that median was compared against the final model pseudo R^2^ value as a means for validating (*p* < 0.05). We performed a log-likelihood ratio test for models with more than one climate index to identify the variable with the greatest log-likelihood value (of NPGO, PDO, or SOI) as the dominant climate index. We interpreted the influence of each quadratic covariate by graphing its relationship with predicted seabird values while controlling for the effects of other covariates in the final models (margins command, STATA 13).

### Habitat Prioritization

We used the conservation prioritization package Marxan (v. 2.4.3, University of Queensland, Australia) to optimize potential nonresident seabird high-use foraging areas to target for potential protection. In Marxan, a ‘reserve system’ describes a set of potential protected areas selected as optimal to conserve [49]. A ‘conservation target’ refers to the percent of the original extent of seabird foraging area (in our research) required for the reserve system [50]. Marxan provides decision support for reserve system design by solving the minimum set problem, for which the objective is to meet the targets of a minimum representation of biodiversity features, at a minimum cost [51]. Using Marxan, we generated scenarios reflecting 10%, 30%, and 50% conservation targets for seabird foraging area in the study region. In our analysis, a 10% conservation target means that Marxan selects the 10% of our predicted seabird foraging distribution that is optimal for conservation. Using the perimeter of the 1-km^2^ grid previously derived for seabird predictions, we computed reserve boundary length. To minimize habitat fragmentation of the targeted reserve area, we calibrated for an appropriate boundary length modifier [52]. Reserve optimizations for n = 100 iterations were generated, each of which gave a count of the number of times each grid cell was included in the optimal reserve solution resulting from each run of the program [53]. The number of times each grid cell was selected for inclusion in reserve design was converted to a percent rank and the upper 50 percent were displayed for 10%, 30%, and 50% conservation target scenarios.

Because global populations of seabirds encounter different levels of stress, we applied a higher species penalty factor to those seabird species whose populations were facing a greater threat of extinction according to the IUCN Red List [54–59]. Using a species penalty factor (spf) enabled Marxan to prioritize some species’ habitat as greater importance for inclusion in the targeted conservation output. We assigned a spf of 1 to species listed as ‘Least Concern’ (northern fulmar, red phalarope, and red-necked phalarope), 2 to those listed as ‘Near Threatened’ (black-footed albatross and sooty shearwater), and 3 to the species listed as ‘Vulnerable’ (pink-footed shearwater).

### Seabird Distribution Visualization and Interpretation

We predicted abundance and distribution for each of the six seabird species to the 1-km^2^ grid cell centroids covering our study area. Maps for each species depict high- to low-use areas by month (May through October) and year (2004–2013). Yearly maps averaged the top three months of peak use (black-footed albatross: May, June, July; pink-footed shearwater, sooty shearwater, and northern fulmar: June, July, September; red-necked phalarope, and red phalarope: July, September, October). To generate a comprehensive map across all six species and across all months and years, we standardized seabird abundance with the following equation:

$${z_{i}=(\frac{x_{i}-\mu }{\sigma })}^{-1}$$ (Eq. 1)

where, *z*_*i*_ is the standardized seabird abundance for grid cell *i*, *x*_*i*_ is the log of each seabird species’ abundance value (*x*) by grid cell (*i*), *μ* is the population mean for that species, and *σ* is the standard deviation, where *μ* and *σ* are log-transformed values. Values were then converted to a percent rank for map display. We generated maps by individual species and for all species standardized and averaged.

Species distributions predicted from final models were compared for spatial difference. The Cramer-von Mises (CVM) test statistic (Ψ) is a modification of the Kolmogorov-Smirnov test that asks whether two populations come from the same distribution [60]. Adapted from Syrjala et al. (1996), seabird abundance values were log-normalized to remove the effect of different population sizes (Eq 2). A script was generated to calculate Ψ (Eq 3; Python v. 2.7.5) for the normalized seabird distributions:

$$p_{ij}=\frac{x_{ij}}{\sum x_{ij}}$$ (Eq. 2)

$$\Psi =\sum {\left(\Gamma p_{i1}-\Gamma p_{i2}\right)}^{2}$$ (Eq. 3)

where *p*_*ij*_ is the log-normalized seabird abundance for log seabird abundance value *x*, grid cell *i*, and species *j*. Ψ ranged from 0 (equivalent distributions) to 0.02 (no overlap of distributions), *p*_*i1*_ and *p*_*i2*_ are two compared log-normalized seabird species distributions (*p*) by grid cell (*i*) and Γ is the cumulative distribution function. Each of the six species was compared to one another and the generalist guild was compared to the planktivorous foraging guild. To determine whether the observed difference between two distributions was statistically significant (*p* < 0.05), we calculated Ψ for n = 1000 randomizations where log-normalized values were randomized for one of the species in the comparison. Before testing with our data, we verified that our CVM script could detect a significant difference (*p* < 0.05) between mock populations with less than 15 percent overlapping distributions. The final p-value was the proportion of the 1000 random Ψ that were greater than or equal to the observed Ψ. For example, if 200 random Ψ were greater than the observed Ψ, then *p* = 0.2, indicating that the difference between distributions was not significant.

## Results

All final models were significant (*p* < 0.05) and successfully validated (k = 10, n = 20, *p* < 0.05; Table 3). We found no multicollinearity between covariates in any of the final models (VIF < 10). The sooty shearwater, red phalarope, and red-necked phalarope were modeled using zero-inflated negative binomial regressions while the black-footed albatross, northern fulmar, and pink-footed shearwater were modeled using negative binomial regressions. Models produced different spatial and temporal patterns depending on species and foraging guild as well as month and year.

**Table 3:** Description of predictive models for distributions of six seabird species.

*Variable*	*Black-footed albatross* *	*Northern fulmar* *	Pink-footed shearwater*	Sooty shearwater*	*Red phalarope* *†*	*Red-necked phalarope* *†*
***Oceanography***						
SSF		Q (-)			L (+)	Q (+)
SSS					L (+)	
SST	**L(+)**			**Q (-)**		
***Bathymetric Features***						
Distance to Cordell Bank	L (-)	L (-)		Q (+)		
Distance to 200-m isobath	**L (-)**		L (-)	Lg (-)	L (-)	L (-)
Depth	L (-)	**Q (-)**	**Q (-)**			
***Terrestrial Features***						
Distance to mainland		L (+)				
Distance to island			Q (-)		Lg (+)	
***Climate Indices***						
UI 3-month lag		Q (-)				
NPGO 1-month lag			L (-)			
NPGO 2-month lag		L (+)			L (-)	
NPGO 3-month lag				Q (+)		
PDO 1-month lag		Q (-)				
PDO 3-month lag	Q (-)			Q (-)	Q (-)	L (+)
SOI current				Q (+)		
SOI 2-month lag	Q (-)					
***Detection Variables***						
Sea state				L (+)		L (+)
Swell					L (-)	
Visibility				L (-)		
Model χ^2^ (df)	517.81 (37)	586.51 (31)	308.55 (26)	654.80 (40)	169.44 (18)	117.10 (15)
Model P value	<0.0001	<0.0001	<0.0001	<0.0001	<0.0001	<0.0001
Model Pseudo-R^2^	0.2251	0.2556	0.1315	0.0970	0.1800	0.0829
Vuong test for zero inflation P				0.0007	0.0324	0.0002
**Model Validation**						
Model Pseudo-R^2^	0.10900	0.3025	0.0128	0.0401	0.1444	0.0185
Validation P value	<0.0001	<0.0001	<0.0001	<0.0001	<0.0001	<0.0001

Strongest relationship of predictor variable from univariate testing is depicted as linear ‘L’, quadratic ‘Q’, or log-linear ‘Lg’. Coefficient value for main effect of predictor variable (i.e. without interaction) in predictive model is depicted as positive (+) or negative (-). Dominant climate index is outlined with a box.

Foraging type is depicted by:

* (generalist) and

† (planktivorous).

Variables exhibiting a significant interaction with year are in bold. Variables represented were significant (*p* < 0.05) in models of each associated seabird species.

### Oceanographic Associations

**Fig 2:**
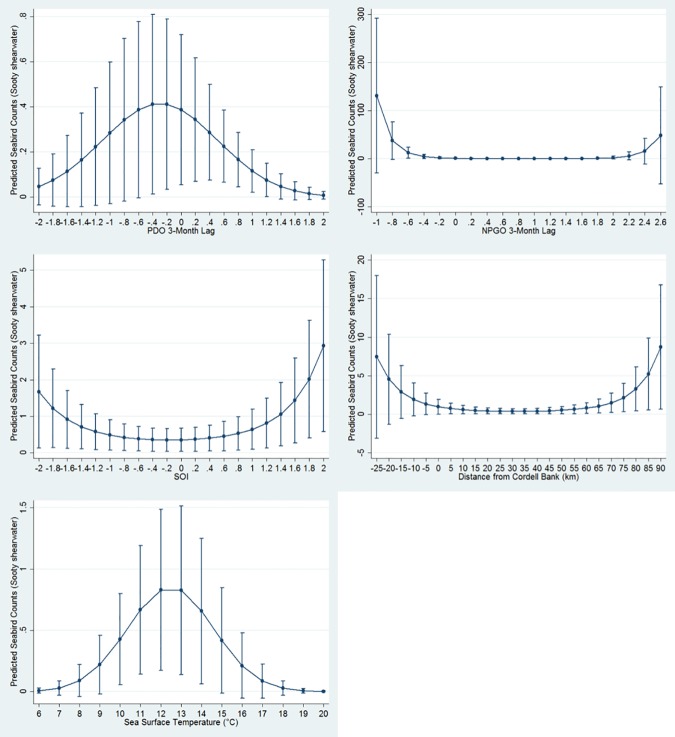
Sooty shearwater quadratic relationships with PDO, NPGO, SOI, distance to Cordell Bank, and SST. Graphs display estimated mean seabird count (y axis) at PDO, NPGO, SOI, distance to Cordell Bank, or SST measurements (x axis). Bars depict +/- 1 standard error.

**Fig 3:**
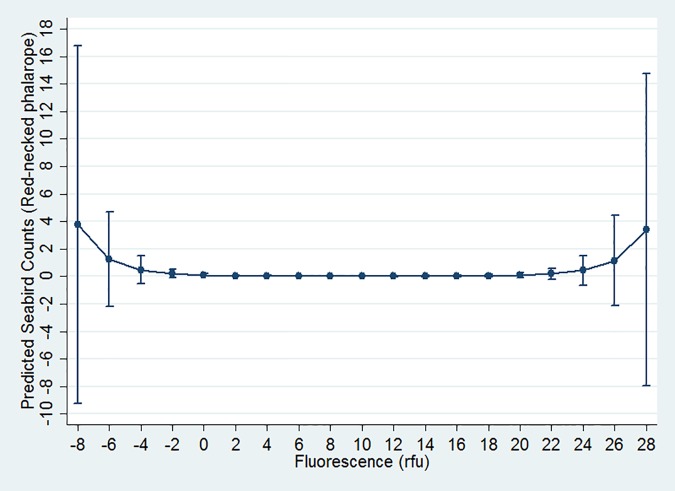
Red-necked phalarope quadratic relationship with SSF. Graphs display estimated mean seabird count (y axis) at fluorescence measurement (x axis). Bars depict +/- 1 standard error.

**Fig 4:**
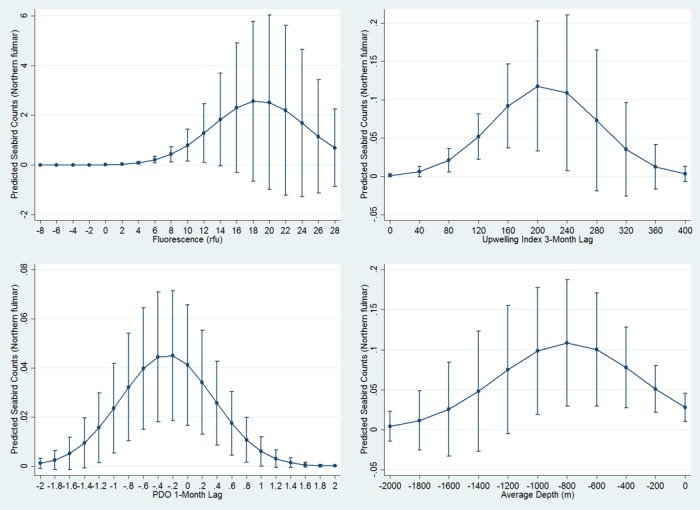
Northern fulmar quadratic relationships with SSF, UI, PDO, and depth. Graphs display estimated mean seabird count (y axis) at UI, PDO, or depth measurements (x axis). Bars depict +/- 1 standard error.

At least one oceanographic variable was important for five seabird models, though no single oceanographic variable was important for the majority of models. Predicted abundance of the sooty shearwater peaked between 12–13°C SST; however, the effect of SST on seabird distribution differed by year (Table 3, Fig 2). The black-footed albatross was positively associated with SST (more seabirds in warmer waters), and the effect of SST also differed by year (Table 3). Only generalist species were significantly associated with SST while only one planktivorous species, the red phalarope, was associated with SSS (Table 3). High abundances of species visiting from Arctic waters were associated with high SSF: both the red phalarope and red-necked phalarope increased for extreme values (Table 3, Fig 3), while the northern fulmar peaked in number at approximately 20 relative fluorescence units (rfu; Fig 4).

### Bathymetric and Distance-Related Associations

**Fig 5:**
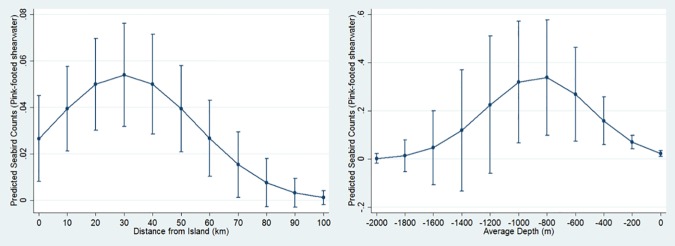
Pink-footed shearwater quadratic relationships with Southeast Farallon Island and depth. Graphs display estimated mean seabird count (y axis) at distance to Southeast Farallon Island or depth measurements (x axis). Bars depict +/- 1 standard error.

Seabird distributions were significantly associated with bathymetric features in all models and, in four models, two or more bathymetric features were significant. For five models, seabird distributions were associated with the 200-m isobath (Table 3). Only the sooty shearwater showed a log-linear association with the 200-m isobath (Table 3), for the other four species, the relationship was linear. The association of the black-footed albatross with the 200-m isobath was significant but varied by year (Table 3). The black-footed albatross and northern fulmar were positively associated with Cordell Bank (the closer in proximity, the more birds). Though the sooty shearwater had a slightly positive association with waters directly over Cordell Bank, a greater proportion of this species’ abundance was associated with distances far from the Bank (e.g. approaching Monterey Bay National Marine Sanctuary; Table 3, Fig 2). The black-footed albatross, northern fulmar, and pink-footed shearwater were negatively associated with depth (Table 3). However, the effect of depth differed by year for northern fulmar and pink-footed shearwater, whose distributions both peaked in abundance at a depth of approximately 800 m (Figs 4 and 5); hence at depths less than or greater than 800 m, abundance decreased.

Seabirds were generally associated with greater distances from the mainland and from islands: northern fulmar and red phalarope abundance, respectively, increased linearly from the mainland and exponentially from Southeast Farallon Island (Table 3). The quadratic relationship of the pink-footed shearwater with distance from islands (Table 3) peaked at approximately 30 km (Fig 5). Only generalist species and not planktivorous species were associated with depth and distance to Cordell Bank.

### Climatic Index Associations

**Fig 6:**
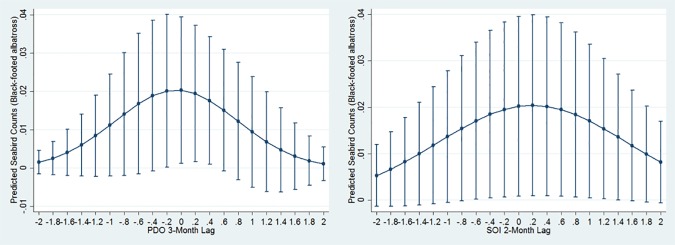
Black-footed albatross quadratic relationships with PDO and SOI. Graphs display estimated mean seabird count (y axis) at PDO or SOI measurements (x axis). Bars depict +/- 1 standard error.

**Fig 7:**
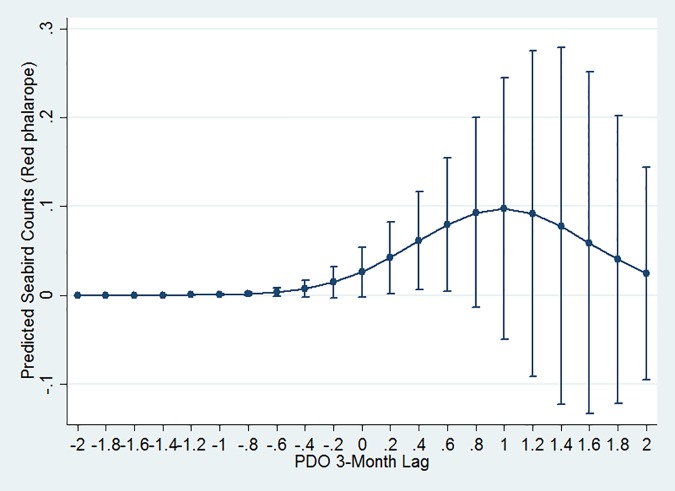
Red phalarope quadratic relationship with PDO. Graphs display estimated mean seabird count (y axis) at PDO measurements (x axis). Bars depict +/- 1 standard error.

All seabirds were significantly associated with climate indices at multiple time lags. We found that generalists each showed quadratic relationships peaking at neutral or slightly negative PDO with either a 1- (northern fulmar) or 3- (sooty shearwater and black-footed albatross) month lag (Figs 2, 4 and 6). Conversely, high numbers of both planktivores (red-necked phalarope and red phalarope) were associated with positive PDO with a 3-month lag (Table 3, Fig 7). Two species from the Arctic were associated with NPGO with a 2-month lag: the red phalarope distribution was associated with a negative NPGO, while the northern fulmar distribution was associated with a positive NPGO (Table 3). In addition, high abundances of two generalists visiting from the South Pacific were negatively associated with NPGO with a 3-month lag (sooty shearwater) or a 1-month lag (pink-footed shearwater) (Table 3, Fig 2). High abundance of two generalists were associated with positive SOI in the month of observation (sooty shearwater) and neutral SOI with a 2-month lag (black-footed albatross) (Table 3, Figs 2 and 6). High abundance of the northern fulmar was associated with a high UI with a 3-month lag (Fig 4).

### Species and Guild Distribution Differences

**Fig 8:**
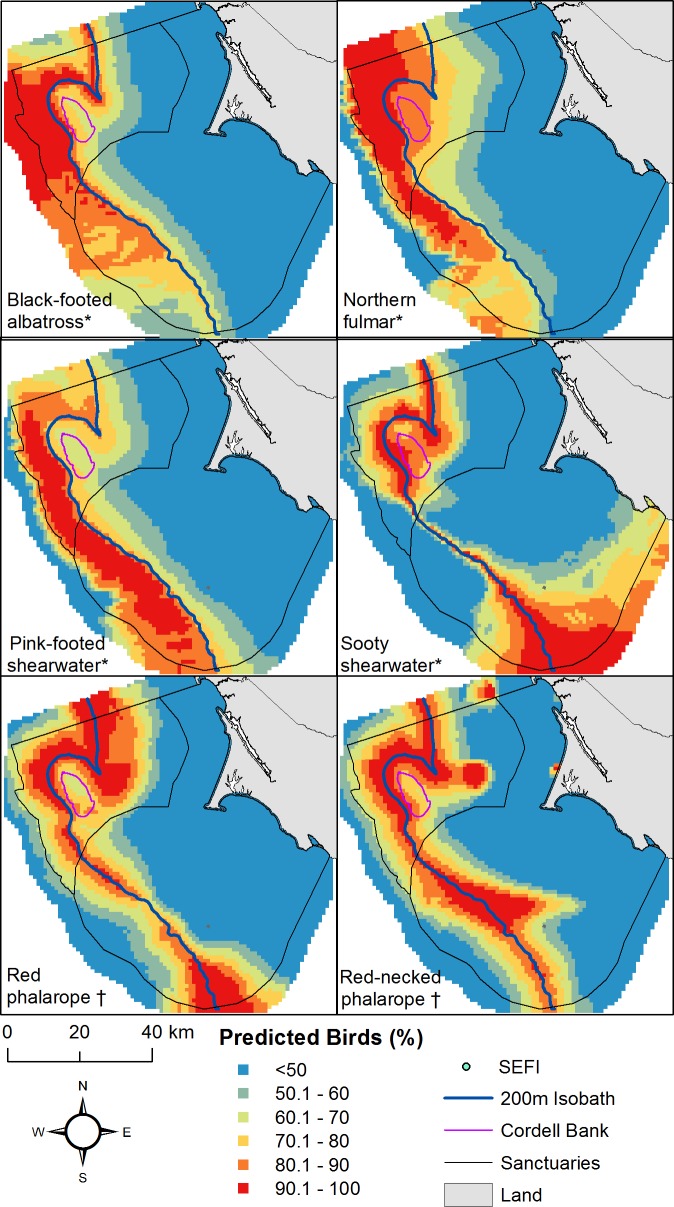
All seabirds standardized and averaged for each species across all years (2004–2013) using predicted values from the top three months of greatest abundance between May and October for each species. Top months were: May, June, July (black-footed albatross); June, July, September (northern fulmar, pink-footed shearwater, sooty shearwater); July, September, October (red phalarope, red-necked phalarope). Map represents highest use foraging areas (upper 50 percent) for each species independent of other species. Foraging type depicted by * (generalist) and † (planktivorous).

**Fig 9:**
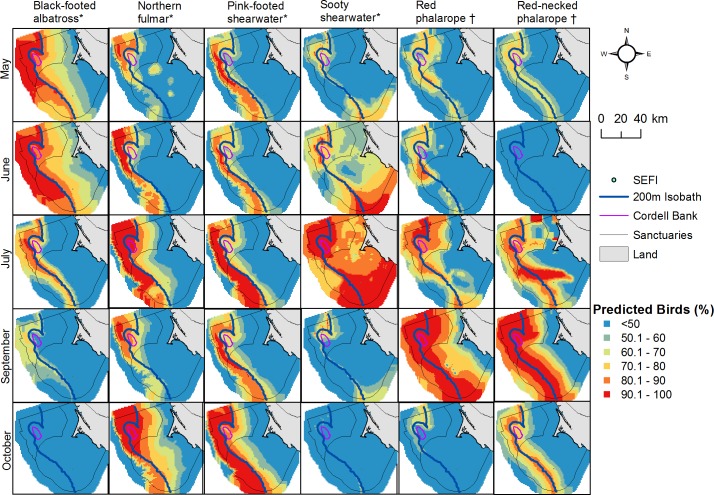
Monthly seabird distributions. Each species was standardized across months (May-October) using predicted values for all months in this range. Map represents highest use foraging areas (upper 50 percent) for each species relative to month but independent of other species. Foraging type depicted by * (generalist) and † (planktivorous).

**Fig 10:**
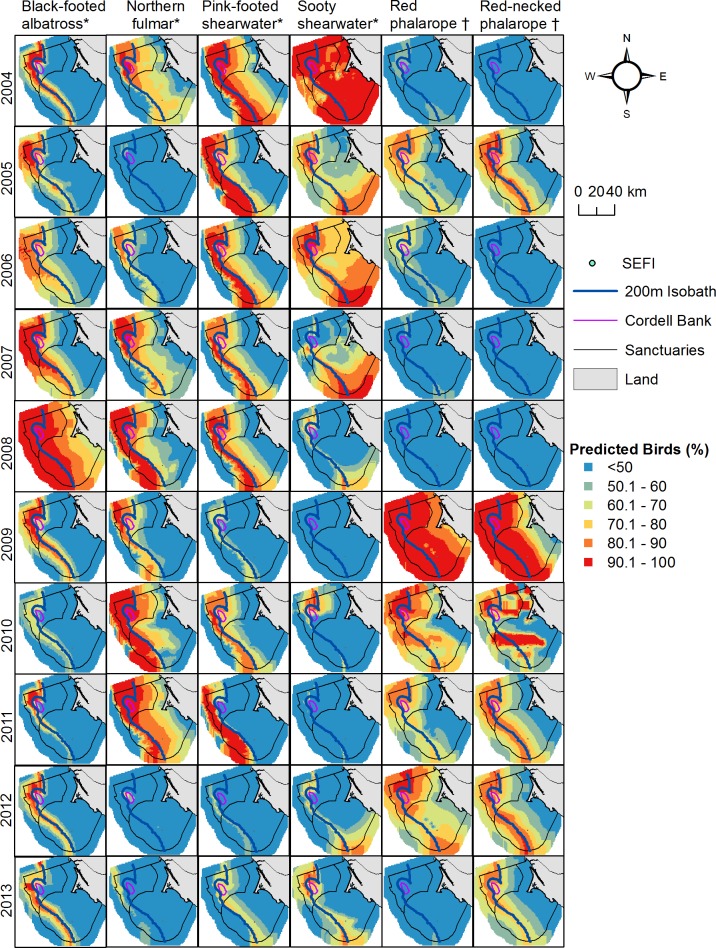
Annual seabird distributions. Each species was standardized across years (2004–2013) using predicted values from the top three months of greatest abundance between May and October. Top months were: May, June, July (black-footed albatross); June, July, September (northern fulmar, pink-footed shearwater, sooty shearwater); July, September, October (red phalarope, red-necked phalarope). Map represents highest use foraging areas (upper 50 percent) for each species relative to year but independent of other species. Foraging type depicted by * (generalist) and † (planktivorous).

Maps of modeled seabird abundances differed by individual species and by foraging guild. Seabirds were most clearly associated with the 200-m isobath when comparing species distributions standardized across all months and years of the survey (Fig 8). Highest predicted use by the black-footed albatross was in June; the northern fulmar, pink-footed shearwater, and sooty shearwater showed a peak in July, though with some variability; and the red phalarope and red-necked phalarope peaked in September (Fig 9). Predicted generalist species use declined while predicted planktivorous species use dramatically increased in the year 2009 (Fig 10). The overall differences between distributions of individual seabird species were not significant except for the sooty shearwater, which was significantly different (*p* < 0.05) from all others (Table 4). Generalist and planktivorous species abundances were associated with many of the same areas and the differences between their distributions were not considered significant (Table 4).

**Table 4:** Species distribution differences characterized by the Cramer-von Mises test statistic ‘*Ψ*’.

Species / *Ψ*	*Black-footed albatross* *	*Northern fulmar* *	Pink-footed shearwater*	*Red phalarope* *	*Red-necked phalarope* †	*Sooty shearwater†*
***Black-footed albatross***	***--***	***--***	***--***	***--***	***--***	***--***
***Northern fulmar***	0.000044	***--***	***--***	***--***	***--***	***--***
***Pink-footed shearwater***	0.000053	0.000027	***--***	***--***	***--***	***--***
***Red phalarope***	0.000064	0.000058	0.000090	***--***	***--***	***--***
***Red-necked phalarope***	0.000052	0.000065	0.000057	0.000075	***--***	***--***
***Sooty shearwater***	**0.000161**	**0.000152**	**0.000223**	**0.000052**	**0.000208**	***--***
**Planktivores vs. Generalists**	*Ψ* = 0.000403					

*Ψ* ranges from 0 (complete species distribution overlap) to 0.02 (distributions do not overlap at all). All species and two foraging guilds were compared.

Foraging type depicted by

* (generalist) and

† (planktivorous).

Significant distribution differences are in bold.

### Detection Error

Our modeling approach accounted for errors in detecting birds surveyed under non-ideal observation conditions (i.e. failure to detect birds when present). The three seabird species that fit zero-inflated models were significantly improved by including three of the tested detection variable factors (Vuong test statistic < 0.05). Sea state and visibility contributed to zero-inflation for the sooty shearwater, while swell height and sea state accounted for zero inflation for the red phalarope and red-necked phalarope, respectively (Table 3).

### Habitat Prioritization

**Fig 11:**
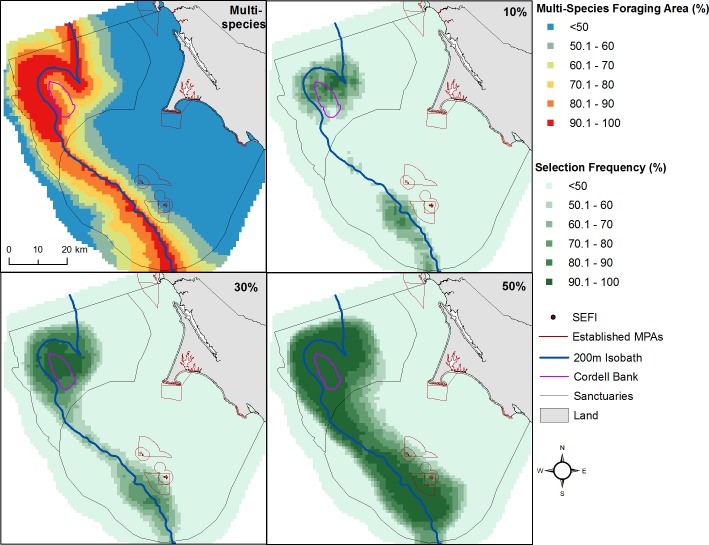
Conservation priority areas. Optimal locations to elevate management status when targeting either 10%, 30%, or 50% of multi-species seabird foraging area. Using IUCN Red List criteria, a higher level of priority was assigned to seabirds with an at-risk level greater than ‘Least Concern:’ pink-footed shearwater (vulnerable), black-footed albatross and sooty shearwater (near threatened).

Our habitat prioritization exercise identified areas reflecting the optimal 10%, 30%, and 50% of seabird foraging area, which encompassed the space most likely to be frequented among the species in this study. Calibration of all scenarios reduced cost and habitat fragmentation thereby increasing the continuity of the conservation area using a boundary length modifier of 10. The three scenarios that satisfied our conservation targets focused on the Cordell Bank vicinity and along the 200-m isobath. The 10% conservation scenario highlighted only an area northwest of Cordell Bank and a southern portion of the 200-m isobath, while 30% and 50% scenarios more fully emphasized the targeting of the full 200-m isobath, including an area radiating out from Cordell Bank (Fig 11).

## Discussion

We found that the associations between predictor variables for nonresident seabirds differed from previous research investigating locally breeding seabirds foraging during the same time frame. Nonresident seabird foraging distributions off the coast of Central California were more sensitive to oceanographic and bathymetric features and less sensitive to distance from islands or the mainland. This finding is in contrast to a previous study that found a strong association between five locally breeding seabirds and distance to their colonies on the Farallon Islands and the mainland [12]. Our use of species distribution models showed that nonresident seabird species models emphasize different predictor variables than models for local species, with the 200 m isobath, in particular, recurring in five of six nonresident species models [12]. Maps generated from these models suggest the foraging areas of nonresident and breeding species differ as well. Though months outside of the April through October period were not in the scope of this analysis, it would be informative to identify where locally breeding seabirds shift their foraging distributions after breeding.

Though oceanographic variables were important for explaining spatial variability in most models, no single variable was important across all models and only one model included two oceanographic variables. Our local scale findings in the GFNMS and CBNMS were similar to findings at the scale of the California Current [7], and indicated that no single oceanographic variable played a key role for all seabirds modeled.

All nonresident seabird species distributions were associated with bathymetric variables. The continental shelf break was prominent as evidenced by the inclusion of the 200-m isobath in the majority of seabird models. This finding verifies the importance of the continental shelf break to seabirds as has been observed in other studies conducted in the California Current [14,15,61–63]; this is also confirmed elsewhere, including areas above undersea canyons near the shelf break off Vancouver Island, British Columbia [64], along the Atlantic shelf break front of austral South America [65], and many other places. We found that several generalist, but not planktivorous, species were associated with the distance to Cordell Bank. While the continental shelf break and Cordell Bank were important bathymetric features, some of the seabird species in our study, particularly the black-footed albatross and northern fulmar, were associated with depths far deeper than the 200-m isobath, similar to that found by Nur et al. [7].

We found that planktivores were associated with the PDO while generalists were not necessarily associated with the same climate index. Only one seabird species, the northern fulmar, was associated with UI, suggesting that the basin-wide climate indices influencing all other species captured most of the variability in local productivity due to upwelling. Northern fulmars were observed in all months during our surveys, but are known to visit the area most commonly during winter months [66]. Our finding that the northern fulmar was associated with upwelling with a 3-month lag may capture the post-breeding dispersal, which coincides with the relaxation of upwelling leading to enough food availability in the months following to support this species as it seeks foraging opportunities south of its breeding grounds. Our finding that climate indices were important for all nonresident seabird models supports recent research showing that climate variability may have increasing ecological significance for seabird foraging activity because of its effects on local oceanography, productivity, and consequently the prey that seabirds seek [22,67].

The abundance of nonresident seabirds was associated with climate conditions 1–3 months prior to their presence in our study area. Associations with a negative PDO indicated that productive conditions 1–3 months prior influenced the abundance of generalists, whereas the association with planktivorous phalaropes was less clear. In our region of the California Current, a negative PDO has been associated with favorable upwelling conditions, strong winds, and typically high productivity [68]. High abundance of phalaropes was associated with a positive PDO, which represents generally unfavorable upwelling conditions in our region of the California Current, but also represents warmer, stratified waters with well-mixed upper ocean layers [68] where fronts are more common [69] and can result in increased foraging opportunities for surface-feeding seabirds [70]. Thus, our results have shown that conditions associated with a negative PDO were linked to nonresident generalist seabirds, which travel to Central California to feed on prey drawn to productive waters, while conditions associated with a positive PDO were more linked to planktivorous phalaropes, which pass through this area and likely stop off in the presence of plankton-rich fronts.

Associations with the NPGO showed that ocean conditions 1–3 months prior to their presence in this region influenced the Arctic species, the northern fulmar and red phalarope and the South Pacific pink-footed shearwater and sooty shearwater. High abundance of the northern fulmar was associated with a positive NPGO with a 2-month lag, a relationship that links this species to upwelling-favorable, productive ecosystem conditions in the Gulf of Alaska and the California Current. Conversely, high numbers of phalaropes and shearwaters were associated with a negative NPGO 1–3 months prior to their presence in this region. A negative NPGO has been shown to represent unproductive conditions, characterized by weak upwelling, and stratified waters with well-mixed upper ocean layers and the presence of fronts [68]. As indicated above, these conditions have been described as conducive to the formation of oceanographic features that are used by birds to find prey near the surface; this includes the phalaropes [71] and also shearwaters [72].

Associations with a positive SOI showed that productive ocean conditions during and up to 2 months prior influenced the abundance of the sooty shearwater and black-footed albatross. A positive SOI has been associated with cold productive waters while a negative SOI has been representative of the more El Niño-like conditions of warm unproductive waters. At the scale of the California Current, Nur et al. [7] found no association with the SOI for the sooty shearwater or black-footed albatross when investigating associations with this climate index at 0–5 month lags, whereas at our more local scale, both the sooty shearwater and black-footed albatross were associated with the SOI.

Beyond the physical features of the shelf break and Cordell Bank that were significant for several seabirds in our analysis, fronts have been characterized as oceanographic phenomena that may further explain predicted seabird foraging distributions. The importance of fronts as areas of plankton accumulation has been well documented [61,73–77]. Associations have been found between predictable, persistent frontal areas and the red phalarope near Cornwall in the United Kingdom [78], the northern gannet (*Morus bassanus*) in the Celtic Sea [79], and the sooty shearwater and the common murre (*Uria aalge*) in the Columbia River plume region offshore from the U.S. states of Washington and Oregon [80]. Frequent occurrence of fronts were found in the vicinity of Cordell Bank and along parts of the shelf break in the Sanctuaries [81]. The locations of prograde (offshore, upwelling-related) fronts in Fontana et al. [81] aligned with our predicted distributions of the planktivorous red-necked phalarope and red phalarope. Thus, our results suggest that the presence of fronts in the Sanctuaries contribute to occurrence of planktivorous species, which feed on accumulated plankton and indirectly contribute to generalist species distributions via their forage fish prey that also feed at fronts.

Our results are consistent with other studies that have used prioritization tools for conservation planning. Previous research using Marxan found that priority foraging areas of locally breeding seabird species in our study region concentrated around and east of the Farallon Islands and northwest of Cordell Bank [12]. Using Marxan, our study has shown that priority nonresident, non-locally breeding seabird foraging area concentrated in the vicinity of Cordell Bank and along the continental shelf break. Interference or exploitative competition [82] between nonresident and locally breeding species may be a contributing factor that could be further explored the former of which has been hypothesized to occur between seabirds and whales [63]. Some of the prioritized seabird foraging area we found fell within established marine protected areas; however, the greater proportion did not.

While banks and the continental shelf break are well-known for concentrating wildlife, it is not always clear what is the most appropriate way to preserve the wildlife in these areas. In the mid-Atlantic, the entire continental shelf break from the 100- to 400-m isobath was recommended for elevated protection status for its harboring of marine species important to commercial fisheries [83]. In addition to Cordell Bank, other banks along western North America’s coast (e.g. Oregon’s Heceta Bank and Alaska’s Bowers Bank) have been recognized as Important Bird Areas (IBAs), which are areas holding a significant proportion of the population of one or more bird species, as identified by repeatedly documented observation of aggregations [84,85]. Though no explicit restrictions on human use or development have been attached to IBA designation, they can provide a starting point for establishing legal status because their establishment requires observational data assessed with standardized global criteria [84]. Recognizing banks as critical foraging areas or fueling stations along migratory routes by elevating their regulatory status would help expand the actively regulated conservation network, or “string of pearls” [86] accommodating seabird migratory paths along North America’s western seaboard.

Important seabird breeding colonies are protected within special wildlife protection zones; however, the protections for critical foraging areas for seabirds are not well-represented [87]. In general, there is a lack of at-sea protected areas because of the difficulties in identifying the critical habitat areas used by highly mobile wildlife and the challenges of effectively managing these sites [88,89]. The approach we have used provides a means to prioritize areas that incorporate multiple species giving highest priority to those species listed as vulnerable or near threatened on the IUCN Red List. Habitat prioritization scenarios as we have implemented provide useful tools to identify critical seabird foraging areas and inform conservation efforts. For example, critical habitats should be prioritized during response activities in case of catastrophic events such as an oil spill or during ocean zoning efforts while considering appropriate location for coastal development for alternative energy. New offshore energy development plans to create wind farms along the California Coast are proposing locations at least 15 miles from shore [90] reaching the continental shelf break in some regions, which, in our study, was among the highest used foraging area for nonresident seabirds. Anticipating potential conflicts between critical habitat and human development in the ocean will minimize negative consequences for marine wildlife.

The global lack of offshore protected areas has been called “the missing dimension in ocean conservation” [87]. Combining resident and nonresident seabird foraging areas will be an important step forward to implementing an elevated management or protective status for the critical areas for seabirds to lead ocean zoning efforts in the Pacific. Identification of high-use foraging areas of multiple species spanning a wider range of marine fauna would complement our findings and provide a more comprehensive picture of key foraging areas to target for management. Further research coupling social and natural systems would be an alternate way to locate areas for special status in global marine reserves and would benefit from assessment of where and when human activities occur in relation to wildlife.
